# Question banks: credit? Or debit? A qualitative exploration of their use among medical students

**DOI:** 10.1186/s12909-024-05517-9

**Published:** 2024-05-24

**Authors:** James Fisher, Declan Leahy, Jun Jie Lim, Emily Astles, Jacobo Salvatore, Richard Thomson

**Affiliations:** 1 Newcastle University School of Medicine, Newcastle upon Tyne, UK; 2 Northumbria Healthcare NHS Foundation Trust, North Shields, UK; 3 School of Medicine and Population Health, University of Sheffield, Sheffield, UK; 4 Newcastle University Medicine Malaysia, Gelang Patah, Johor Malaysia

**Keywords:** Question banks, Assessment, Medical students, Gamification, Self-determination theory, Multiple-choice questions

## Abstract

**Background:**

Online question banks are the most widely used education resource amongst medical students. Despite this there is an absence of literature outlining how and why they are used by students. Drawing on Deci and Ryan’s self-determination theory, our study aimed to explore why and how early-stage medical students use question banks in their learning and revision strategies.

**Methods:**

The study was conducted at Newcastle University Medical School (United Kingdom and Malaysia). Purposive, convenience and snowball sampling of year two students were employed. Ten interviews were conducted. Thematic analysis was undertaken iteratively, enabling exploration of nascent themes. Data collection ceased when no new perspectives were identified.

**Results:**

Students’ motivation to use question banks was predominantly driven by extrinsic motivators, with high-stakes exams and fear of failure being central. Their convenience and perceived efficiency promoted autonomy and thus motivation. Rapid feedback cycles and design features consistent with gamification were deterrents to intrinsic motivation. Potentially detrimental patterns of question bank use were evident: cueing, avoidance and memorising. Scepticism regarding veracity of question bank content was absent.

**Conclusions:**

We call on educators to provide students with guidance about potential pitfalls associated with question banks and to reflect on potential inequity of access to these resources.

**Supplementary Information:**

The online version contains supplementary material available at 10.1186/s12909-024-05517-9.

## Background

Assessment forms a central component of medical students’ progression through medical school. It is therefore unsurprising that assessment acts as a powerful driver for learning and as a source of anxiety for students [1]. Multiple choice questions (MCQs) have become embedded in medical education as assessment tools due to their feasibility, reproducibility and cost-effectiveness [2]. Developing and refining strategies to optimise preparedness for such examinations is therefore imperative to early-stage medical students. The ubiquity of internet access and device ownership amongst medical students [3] has led to a dramatic change in the nature of the resources used to prepare for assessments. A survey of Australian medical students in 2015 [4] identified that online question banks were the most popular education resource, with students using them for revision and for learning new content. Question banks contain a repository of practice MCQs; typical formats include single-best answer (SBA), extended matching questions or true or false questions. Kumar et al. [5] undertook a retrospective cross-section survey of study habits amongst American medical students who were preparing for the United States Medical Licensing Examination (USMLE) Step 1. Strikingly, respondents reported that around half of their study time was spent doing practice questions, with respondents completing a mean of 2,666 questions. There is also some evidence to suggest that the use of online question banks correlates with USMLE performance [6].

Whilst question banks are popular and have the potential to improve assessment performance, there are potential pitfalls associated with their use. First, question bank content may not align with a student’s host institution curriculum. Second, online resources may not be accurate or up-to-date [7], meaning students may inadvertently acquire, or reinforce, erroneous knowledge. Third, many question banks are hosted behind paywalls, introducing potential inequity of access. Financial hardship amongst medical students is increasingly recognised [8] and worries about financial status have been linked to reduced performance in assessments [9].

There is an absence of evidence explaining why question banks have gained such popularity amongst medical students. There is also a lack of literature exploring how medical students incorporate question banks into their learning and revision strategies. Against this backdrop, we will use Deci and Ryan’s self-determination theory to facilitate exploration of this area [10, 11]. Self-determination theory (SDT) provides an explanation for human motivation. It posits that humans are growth-orientated and that three innate psychological needs foster this – autonomy, competence, and relatedness [12]. A sub-theory, organismic integration theory, describes three different regulatory structures that underpin motivation. Amotivation, at one end of the continuum, describes a complete lack of desire. Conversely, intrinsic motivation is recognised as driving engagement with a task through personal interest or satisfaction, and is associated with deeper learning, improved performance and greater well-being [13, 14]. Between these sits extrinsic motivation, where behaviour is prompted by an external locus of control, be it rewards, punishments, or demands that must be achieved. Extrinsic motivation is further divided depending on the extent to which the external regulation of behaviour has been internalised and integrated into one’s own thinking [11].

Our study had two aims: (1) To explore why early-stage medical students choose to use online question banks as part of their learning and revision and (2) To explore how early-stage medical students integrate online question banks into their learning and revision strategies. For the purposes of this research, we defined question banks as extra-institutional online resources produced by profit-making organisations that recreate common assessment formats.

## Methods

### Setting

The study was conducted across Newcastle University Medical School’s two campuses: Newcastle-upon-Tyne, United Kingdom (UK), and Newcastle University Medicine Malaysia (NUMed) in Johor, Malaysia. Students from both campuses are considered one cohort; the same curriculum and assessments are delivered at both sites and students graduate with the same Newcastle University MBBS degree.

### Design

The phenomenon of interest in this research, students’ experiences of using question banks, aligns with an interpretivist paradigm; there is no single, unifying student experience and reality is both dynamic and subjective. We therefore selected qualitative interviews as the method for exploring students’ experiences with question banks. Deci and Ryan’s SDT was selected as a theoretical ‘lens’ through which findings would be interpreted. When reporting our findings, we have adhered to the COREQ criteria for reporting qualitative research [15] (except for participant checking).

### Reflexivity

The research team consisted of six members (one female and five male) with a range of education and clinical experience - two MBBS students (DL&JL), two clinical teaching fellows (EA&JS), and two senior clinical lecturers (JF&RT). All interviews were conducted by EA and JS. EA and JS had taught the UK-based students who participated (and one of the NUMed students who had visited the UK on an exchange). None of the research team held a mentor or supervisory role with participants. The analysis process was led by DL and JL, but all members of the research group provided input into the development of the thematic framework through regular project meetings. These meetings provided a forum for discussion about initial analysis of the data. In these meetings nascent themes could be challenged and alternative interpretations of the data could be offered.

### Sampling and recruitment

Purposive, convenience and snowball sampling of medical students in their second year of study was employed, since all would have sat at least three SBA examinations, which first year students would not have. Later-stage students were not targeted, since in the Newcastle MBBS programme, the emphasis in examinations for later-stage students shifts towards more practically orientated examinations, where question banks may be less relevant in examination preparation. All potential participants were approached via email. Those expressing interest in the project were sent a participant information sheet, which described the study and data management procedures. Those students willing to participate provided informed, written consent.

Inclusion criteria were therefore: second year student on the Newcastle / NUMed MBBS programme, willing and able to provide informed consent to participate. Exclusion criteria were: year of study other than second, unable to provide informed consent.

### Data collection

Interviews were conducted using a semi-structured guide which was piloted during a test interview. This was observed by a senior member of the research team (RT) who provided feedback on interviewing style. Following this, modifications were made to the semi-structured interview guide (Supplementary Material). All interviews were conducted online using Microsoft Teams (13/04/2023-20/07/2023). Online interviews enabled participation of students regardless of geographical location. All interviews were recorded and were then transcribed verbatim (DL and JL). Participants were assigned a number in place of their name to anonymise their data.

A total of ten interviews took place. No participants withdrew consent during the study.

Interviews ranged in duration from 25 to 59 min (mean = 46, median = 48). Field notes made during interviews were collected in physical and electronic formats and were circulated to the research team via email.

### Data analysis

**Fig. 1:**
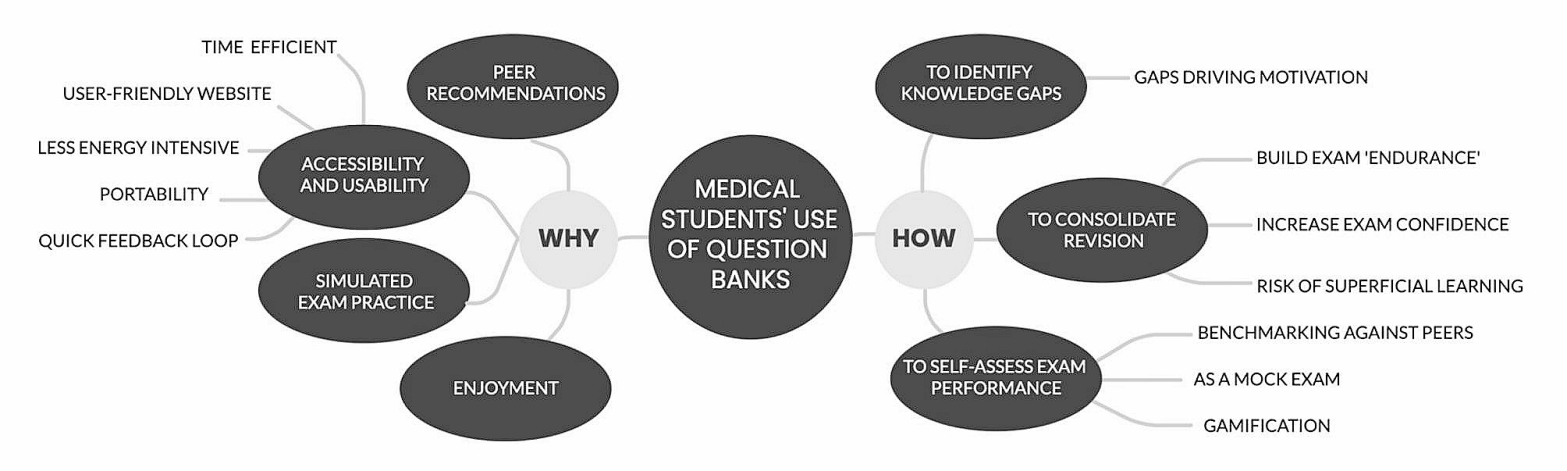
Overview of themes and sub-themes derived from analysis

All interview transcripts were uploaded into NVivo13. Thematic analysis was undertaken, in line with the six-stage process outlined by Braun and Clarke [16]. DL and JL read and re-read the interview transcripts to familiarise themselves with the data. All transcripts were dual-coded by both DL and JL, who held regular meetings to compare coding sets. Considerable overlap was apparent, but there were instances of differing interpretation - in most instances these were resolved by DL and JL and the changes were incorporated into the coding set. Where discord remained, discussion was held amongst the wider research team and consensus was reached. Analysis was performed iteratively after each interview. Modifications were made to the semi-structured interview guide iteratively during the analysis phase of the study (after interviews 3 and 7), to enable exploration of nascent themes. Codes were organised into over-arching themes and sub-themes (Fig. 1). There was no a priori assumptions about themes, which were based solely on analysis. Data collection ceased when no new participant perspectives were identified from the data.

#### Ethics statement

Ethical approval was received from Newcastle University School of Medicine Research Management Group on 23/11/22 and the University Ethics Committee (Ref: 2289/18,929).

## Results

### Participant demographics

Of the 10 study participants, 5 were students at the NUMed campus and 5 were UK-based (Table 1).

**Table 1:** Study participants

Interview	Duration (mins: secs)	Campus
1	59:46	UK
2	56:55	UK
3	47:35	NUMed
4	39:29	NUMed
5	52:45	UK
6	56:50	NUMed
7	49:53	NUMed
8	40:03	UK
9	40:22	NUMed
10	25:58	UK

### Why do students use question banks?

#### Peer recommendations

Participants described how peers in senior year groups had made them aware of online question banks as a key resource for successful progression through medical examinations.

*“Everyone has said positive things about them… if older years are saying this, and they’re going through medical school, perhaps it’s a factor that can allow you to do better in exams as well.” [#2]*

Participants envisaged making similar recommendations to students in more junior year groups. Such recommendations were underpinned by a belief that question banks ‘worked’, with students directly citing their use as the reason for improved academic performance:

*“I just got my results and my SBA performance has improved from 58.1–68.9% - I could confidently say that using the question bank in semester 2 greatly improved my performance.” [#5]*

#### Simulated exam practice

Assessment was described, universally, as a stressor. Fear of exam failure, and potential removal from the course, was a potent motivator.

*“What I fear the most is that I fail and have to leave the university”* [#6]

The use of question banks filled perceived gaps that made participants feel uneasy. Participants wanted access to greater numbers of practice questions – whilst they received some within the course, this was felt to be insufficient. Participants cited examples of how, during school-level education, ‘past papers’ formed a valuable resource for exam preparation. Greater exposure to exam questions helped participants to develop their exam technique, through greater familiarity with the phraseology and question styles.

*“Question banks are more like simulation – even if the questions are not that relevant, you have practiced before, so you’ve got trained with this style – so in the main exam I feel much more confident.” [#5]*

There were suggestions that students’ host institutions could provide their own question bank, but these were tempered by the realisation that this may not be feasible due to insufficient numbers of questions being available.

#### Accessibility and usability

Participants described a sense that engagement with question banks did not require a significant investment of energy or concentration, meaning students were drawn towards using them when they felt unable to focus on other resources:

*“The question bank helps me when I’m not really in the mood to focus on the lecture slides and my own notes – just selecting options… gets me motivated again and keeps me going” [#6]*

Similarly, students considered question banks as an antidote for procrastination.

*“We use questions when we don’t want to study and we know that we’re not being productive in the day – we just… try to solve as many as we can… to make sure that we do something while procrastinating.” [#9]*

Students valued the speed at which feedback could be obtained. Comparison was drawn between the time it would take to determine whether an answer was correct or incorrect using a textbook. Question banks were therefore felt to be a time-efficient method for learning.

*“I feel like you don’t need to invest too much time to get a lot of knowledge out of question banks” [#8]*

The ability to access question banks on smartphones and internet-enabled devices rendered the resource far more portable and thus convenient.

*“It’s the convenience – you can use them anywhere, like on train – they’re so much more convenient than books” [#10]*

Participants described registering for question banks to take advantage of a free trial but having used them, being willing to pay for access in future.

*“I was of the idea that I would not pay before I started using them, but now I have used them, it’s became an essential part of my studies, so I think I would pay for them in the future.” [#10]*

The visual appearance of the website, and the functionality of its user interface, appeared to directly influence student’s perceptions of the quality of the resource and the veracity of the materials.

*“It has a very furnished and pristine website, which gives me implicit trust in the questions” [#1]*

There was a widespread lack of understanding about who produced the questions and the process by which they were developed.

“*I’m not entirely sure how the questions were made. I assume there’ll perhaps be doctors and older medical students who chipped in and then it gets checked by clinicians…” [#2]*

#### Enjoyment

Some students described question banks as being enjoyable, citing how their interactivity helped to stave off boredom. Similarly, some students valued question bank content that linked to clinical situations, as this helped them envisage the relevance of their learning to future clinical scenarios.

Some students referred to in-built mechanisms within question banks that rewarded progression. These moved beyond simply keeping a count of correct and incorrect responses and provided visual rewards for the number of questions completed. The act of being rewarded, despite it having no tangible, intrinsic value, appeared to function as a motivation to continue learning.

*“Each time we get a question right, we see a certain allocation of points… when (you) get a certain amount of points… (you) feel like continuing a bit more”[#6]*

### How do students use question banks?

#### To identify knowledge gaps

Students considered question banks an effective method to identify gaps in their knowledge.

*“Through questions I know what I know, and I know what I don’t know.” [#7]*

There was however variation in the time when question banks were used. Some described using them mid-term, typically after completing a module of learning. Search functions within question banks, to restrict questions posed to specific topic areas, allowed targeted evaluation of knowledge. Some described using them at the outset of their revision to determine their overall knowledge and as a signpost to where they ought to focus subsequent learning.

*“Getting questions wrong tells me there’s more to work on, so I need to study more – I actually make notes from the question I got wrong.” [#3]*

Students described times where they would deliberately use question banks to provoke this fear, and thus spark motivation.

*“If I’m feeling very lazy or very demotivated then I’ll just do questions… a lot of the time, once you’ve done questions, you’re either scared or motivated.” [#4]*

#### To consolidate revision

The use of question banks was usually a solo pursuit. Some students described collaborative use whereby students would congregate in-person or online to work through questions together, whilst discussing answers and uncertainties.

Intensity of question bank use increased as assessments grew closer. The large volume of questions available within such resources was valued and used to build exam “endurance”.

*“There was like 700 questions… so I just stayed at home… and just answered as many as I can.” [#10]*

Students envisaged question banks as a method by which their revision could be enhanced, and drew analogies to physical training:

*“It’s more a supplement, like how some gym goers like to use protein powders… I guess for medical students, question banks act like supplements…. bulk up with the knowledge” [#2]*

There appeared to be an addictive element to question answering, particularly when correct responses were selected:

*“Sometimes when (you) are doing the questions and keep getting them right, (you) don’t feel like leaving and want to continue.” [#6]*

However, the large volume of questions, coupled with a perceived need to ‘complete’ the question bank was detrimental for some.

*“I think it [the need to finish all the questions] is bad in a way – things can get very overwhelming and cause you to have all kinds of anxiety, especially pre-exam.” [#3]*

In contrast, some found that stress levels were reduced by completing large numbers of questions, since doing so provided a sense of reassurance that there would be no “surprises” in the actual examination. For some, the volume of questions, coupled with the perceived need to review them all, resulted in completion of the questions becoming the primary focus and acted as a distraction from meaningful engagement:

*“[you] just do the questions… but you haven’t fully understood it – you’re just doing it for the sake of it*.” [#6]

Students described how the nature of the questions themselves were also sometimes a distraction to deeper learning. There was a sense that question stems that recurred could be recognised and completed successfully through “memorisation”, without the need for deeper understanding. Furthermore, students described how the visibility of the potential answers within MCQs was contrary to real-world clinical decision-making and over-simplified the process of clinical reasoning:

*“They always give options, so we rely on the options to eliminate or to choose. When it comes to the application of it in the hospitals, no one gives you options… you have to figure it out from like a million other diseases.” [#9]*

#### To self-assess exam performance

Some students used question banks as a form of mock examination after completion of their revision.

*“I’ll go through all my notes and then use the question banks as practice to see how much I actually learned.” [#8]*.

There was however some concern that the question bank content (topics or difficulty level) and the question style, may not align with that of their local institution. Question banks provided students with a visible, quantifiable, real-time measure of their knowledge based on their performance with questions they had completed. Some students identified a pre-test score that they then targeted, tracking their performance over time to “chase” that score. Some question banks also provided the average score achieved by all platform users, thus enabling benchmarking against one’s peers.

*“The analytics of seeing your performance over time is really good, also comparing your performance with the average user, kind of gauging a sense of how you’re doing.” [#2]*

Whilst some students deliberately ignored the score, thus avoiding unwanted comparisons with peers, others found such comparisons motivating. For some though, this ever-present performance metric was stress-inducing, particularly if their score fell below that of platform users or below a known, or assumed, pass-mark.

*“If I got very low, for example if I got below 50%, I would be stressed.” [#3]*

There was also evidence that some students placed such focus on maintaining their score that it detracted from potential learning opportunities. There were instances where students in effect manipulated the score, through selective completion of questions:

*“Sometimes you want to compare your score or percentage with other people, and then it’s not really learning, I’d skip the hard questions just to get a 90% as opposed to an 80%.” [#1]*

Performance data was also available for individual questions. For example, when a student answered a question incorrectly, and then discovered that the most platform users had answered correctly, this provided a clear warning to them that their understanding of that topic required development.

## Discussion

Students’ motivation to use question banks was overwhelmingly driven by extrinsic motivators, with high-stakes exams and fear of failure being central. Due to their convenience and perceived efficiency, students deliberately turned to question banks during periods when they were tired or lacking in drive, or when they felt they ought to be doing more work. In such situations, their use often highlighted deficiencies in knowledge, provoked feelings of anxiety and guilt and generated a potent external motivation to work. This finding aligns with introjected regulation, a regulatory style within extrinsic motivation, where there is partial internalisation of the regulation of behaviour, but it is not truly accepted as one’s own - it is often guided by a desire to reduce guilt and promote self-esteem [17]. Intrinsic motivation by contrast, was less evident, with limited reference made by students to engagement with question banks due to curiosity and enjoyment.

Students became aware of question banks through conversations with senior peers, who identified them as being essential for overcoming assessment hurdles. Students were strongly influenced by these recommendations and described how they too would recommend them to more junior colleagues. This exemplifies how the educational milieu in which a student learns, and specifically their relationships with peers, influences motivation through the process of internalisation. ‘Significant others’ are recognised as being potential catalysts for the creation of introjected modes of motivation by externally controlling it with rules – ‘it’s what you need to do to pass’ [12]. Students in differing year groups share ‘social congruence’ – the sharing of similar social roles and a familiarity with the local – this has been recognised as promoting transition of knowledge between cohorts [18]. Mentoring, exhibited informally here, has also been recognised as a stimulus for intrinsic motivation [19]. Despite question banks being a primarily solitary pursuit, there was some evidence of collaborative learning when using them, both in-person and remotely. This encouraging environment engendered a sense of relatedness and thus promoted intrinsic motivation. However, we contend that the scoring systems within question banks, and the strong urge to compare scores with one’s peers, significantly undermined this relatedness and thus stifled intrinsic motivation.

The portability and convenience provided by question banks, which could be accessed by students on personal devices at any time of their choosing, tapped into the psychosocial need for autonomy described by SDT, by giving students complete control as to when their knowledge would be tested. The ability to gain an immediate judgement on one’s knowledge provided students with a metric by which their learning could be evaluated. This engendered a sense of self-efficacy and provided a stimulus for intrinsic motivation. It was however striking that there was little or no scepticism as to the veracity of the answers provided by question banks.

Students strove for competence, a core psychological need described by SDT, through repeated testing, spaced out over time. Recalling previously learnt information enhances the ability to recall the information in future – this, in the context of a test, is referred to as test-enhanced learning [20]. The pattern of learning inherent to question banks - answering a question and then immediately receiving the answer, generates rapid feedback cycles, which is a feature recognised within the literature on gamification as maintaining engagement [21]. Gamification describes the process by which designers use “game-based mechanics… to engage people and motivate action” [22]. However, rapid feedback cycles are linked with compulsive internet use [23] and for some students there were suggestions of such engagement patterns. For some, the need to complete the entire repository of questions became overwhelming and anxiety-provoking. Virtual reward structures present within question banks (e.g. scoring, competition) are also recognised as drivers for addictive and compulsive engagement [24] and there is strong evidence that rewards act to diminish intrinsic motivation [25].

Other potentially detrimental patterns of question bank practice were evident. First, cueing. This is a situation where a learner is able to answer a question through recognition of the correct option; however, in the absence of that option, the learner would not be able to answer correctly [26]. Second, avoidance. Here, the element of competition, in terms of their question bank score, was such a potent source of extrinsic motivation that students deliberately avoided more challenging questions to avoid ‘damaging’ their score. This would be considered in the gamification literature as an ‘undesired behaviour’ produced by the gaming element [27]. In this context, such behaviour highlights that the focus of the learner is primarily on the gamification mechanic as opposed to the learning itself and exemplifies how extrinsic reward systems can act to undermine intrinsic motivation [28, 29]. This also aligns with introjected regulation - the goal of improving one’s score is been identified as being personally important to the student, such that it drives their behaviour.

The final, potentially detrimental pattern of question bank use was memorising. Repeated practice meant increasing familiarity with the question banks, such that students often learnt what the right answer was, without necessarily holding a deeper understanding as to why. Memorisation of factual knowledge, without deeper understanding, was both quicker and more productive in terms of question bank performance. This pattern of use may have been more apparent within our study, since previous work has outlined how NUMed students believed their experiences within the Malaysian education system placed greater emphasis on memorisation of factual knowledge [30]. Through question bank use students became conditioned, through repetition and repeated feedback, to identify the ‘usual’ answer when particular clinical descriptions or wording appeared in the question stem. Such patterns of learning are potentially problematic when learning about clinical medicine and diagnostic reasoning, since premature closure of thinking (failing to consider alternatives), is recognised as a significant contributor to diagnostic error [31]. This issue may also be compounded by the nature of MCQs themselves. Constructing valid MCQs that are rich in context, containing ambiguities and dilemmas, is extremely difficult and may result in the potential for examination of minutiae [32].

### Strengths and limitations of the research

A strength of this work was the rigorous, iterative approach that was applied to analysis. This enabled refinements to be made to the interview schedule over time and for nascent, unexpected themes to be pro-actively explored. The use of SDT as a theoretical framework helped inform both study design and data analysis and ultimately, helped advance understanding of the phenomenon of interest. A further strength is the breadth of experience levels that our diverse research team brought to the project. Whilst participant checking of findings was not undertaken, the central involvement of medical students within the research team ensured that the findings were grounded in their lived experiences of the course, and as a result enhanced their authenticity.

## Conclusion

Our findings demonstrated that students’ motivation to use question banks was predominantly driven by extrinsic motivators, with high-stakes exams and fear of failure being central. Their convenience and perceived efficiency promoted autonomy and thus motivation. Rapid feedback cycles and design features consistent with gamification were deterrents to intrinsic motivation. Potentially detrimental patterns of question bank use were evident: cueing, avoidance and memorising. Scepticism regarding veracity of question bank content was absent.

Medical educators must acknowledge that the use of question banks amongst medical students is widespread and, as our work shows, they form an integral part of students’ learning and exam preparation. Our research focussed on early-stage medical students, so future research might explore how later-stage students, or healthcare professionals undergoing post-graduate examinations, use question banks within their educational development.

We call on educators to provide students with specific guidance about potential pitfalls associated with online question banks. Students should be made aware of the potential for question banks to engender unhelpful learning strategies and to promote superficial learning. Furthermore, students should be made aware of the skew that can be applied to one’s learning when question banks are used extensively. For example, it is recognised that the patients described within question banks are not reflective of the diversity of society [33]. We commend Grinberg, writing in JAMA [34] - her powerful narrative illustrates how reductionist MCQs can be and how they can stifle empathy amongst clinicians.

In view of the absence of any scepticism as to the veracity of question banks, students ought to be taught digital curation skills early, with the aim of encouraging more critical engagement with question banks as learning resources. This caveat is offered in the context of the imminent, inevitable encroachment of Artificial Intelligence on the world of assessment, with question banks likely to be early adopters [35]. Given their widespread use, educators ought to consider potential inequity of access to question banks amongst their learners. Supporting students to develop their own questions [36, 37] may overcome this and may also enable closer alignment with local curriculum whilst embedding quality control and governance processes. Lastly, educators may also wish to reflect on whether MCQs are the optimal assessment method in a world where medicine is increasingly complex and nuanced.

### Electronic supplementary material

Below is the link to the electronic supplementary material.

Supplementary Material 1

## Data Availability

Individual interview transcripts have not been made available to maintain the confidentiality of study participants. The output of the analysis derived from these interviews is presented in full in the manuscript. For queries in relation to the data analysis derived from this study, please contact james.fisher@ncl.ac.uk.
