# Supplementary material for: Question banks: credit? Or debit? A qualitative exploration of their use among medical students
Source: BMC Med Educ. 2024 May 24;24:569. doi: 10.1186/s12909-024-05517-9 (PMC11127331; doi:10.1186/s12909-024-05517-9)
Supplement: Supplementary file 1 — Supplementary Material 1 [file 12909_2024_5517_MOESM1_ESM.docx]

**Supplementary Material: Semi-structured interview guide**

**Introduction**

- Introductions, review of consent and opportunity for questions
- Reminder of audio-recording (thereafter recoding starts)
- Explanation of the aim of the project: to explore early-years medical students’ motivations for using QBs, perceptions of the utility of QBs, understanding of pitfalls associated with QBs, and thoughts on future QB usage.

**Questions:**

- Tell me about all of the resources you use to learn and revise
- Where did you first hear about question banks?
  - Have you spoken to more senior medical colleagues with regards to the use of question banks?
- What question banks do you use?
  - What influences your decision?
- How do you use question banks them?
  - When in the year?
  - Where?
  - How long?
  - Who with – solo / with peers?
  - Are you targeting a specific percentage?
  - What do you do when you get a question wrong?
  - Where do question banks fit within your learning and revision (compared to other resources)?
- Question banks – why do you use them?
  - What motivates you to use them? What are you trying to achieve?
    - Exam technique? Benchmarking self /comparing to others
  - What do you get out of using them?
  - Why question banks and not other learning resources?
- Question banks – feelings
  - Do you compare your scores to peers?
  - Do you track your scores against the user average?
  - How do you feel if you get a question wrong?
- What are some of the pitfalls of using question banks?
- Do you have any concerns about using question banks?
  - Do you trust question banks?
  - Do you know who writes the questions?
  - What do you do if you think a question is wrong?
- Do you pay for / would you pay for question banks?
- Do the question banks align with the curriculum?
  - Which areas of your learning do question banks help with?
  - Which exams do you find question banks helpful for?
- What are your top tips for first years coming into second year with regards to using question banks?
- Are there any other comments you would like to make related to the interview that we have not discussed?
